# Synthesizing 2D MoS_2_ Nanofins on carbon nanospheres as catalyst support for Proton Exchange Membrane Fuel Cells

**DOI:** 10.1038/srep28088

**Published:** 2016-06-15

**Authors:** Yan Hu, Daniel H. C. Chua

**Affiliations:** 1 Department of Materials Science and Engineering, National University of Singapore, 9 Engineering Drive 1, Singapore 117575.

## Abstract

Highly dense 2D MoS_2_ fin-like nanostructures on carbon nanospheres were fabricated and formed the main catalyst support structure in the oxygen reduction reaction (ORR) for polymer electrolyte membrane (PEM) fuel cells. These nanofins were observed growing perpendicular to the carbon nanosphere surface in random orientations and high resolution transmission electron microscope confirmed 2D layers. The PEM fuel cell test showed enhanced electrochemical activity with good stability, generating over 8.5 W.mgPt^−1^ as compared to standard carbon black of 7.4 W.mgPt^−1^ under normal operating conditions. Electrochemical Impedance Spectroscopy confirmed that the performance improvement is highly due to the excellent water management of the MoS_2_ lamellar network, which facilitates water retention at low current density and flood prevention at high current density. Reliability test further demonstrated that these nanofins are highly stable in the electrochemical reaction and is an excellent ORR catalyst support.

MoS_2_ is a promising material in the energy conversion field with its layer structure that similar to graphene. Inspired with the success of few-layer or single-layered graphene, these transition metal dichalcogenides have received increasing attention due to their potential for a range of applications. Unlike the highly conductive graphene, MoS_2_ is a direct bandgap semiconductor allowing fabrication of semiconducting and optoelectronic devices. In the field of clean energy, previous studies have shown its good catalytic activity in hydrogen evolution reaction (HER) in various types of and with different supports^1^,^2^,^3^,^4^,^5^,^6^. Among various reports on HER efficiency, researchers have reported that MoS_2_ is able to catalytically do water splitting^6^ and several others further show MoS_2_ as a catalyst support for various solid-oxide fuel cell work. In the case of the latter, it has been further shown to be resistant to carbon monoxide poisoning hence improving the overall effectiveness^7^.

However, in the field of proton exchange membrane fuel cells (PEMFCs), the role of MoS_2_ in oxygen reduction reaction (ORR) is less studied compared to the extensively studied graphene or carbon nanotubes^8^,^9^,^10^. In 2013, Wang *et al*.^11^ reported the size-dependent ORR activity among a group of ultrathin MoS_2_ nanoparticles that were obtained by an integrated method of sonication and centrifugation. Their results indicated that the enhanced ORR current density corresponded to the increasing amount of Mo edges with smaller particle size; hence the best performance was produced by the 2 nm MoS_2_ particles that favored the four-electron ORR route. In their subsequent work^12^, AuNP/MoS_2_ films, which were synthesized by a simple drop-casting modification process, showed the enhanced catalytic activity and superior stability than the commercial Pt/C catalyst, and the improvement was explained by the synergetic catalytic effect of Au in the onset potential and MoS_2_ in the ORR 4-electron kinetic. Nevertheless, their ORR measurement was undertaken in a simulated alkaline fuel cell environment instead of a real PEM fuel cell. There is still little investigation to date about the activity of MoS_2_ as catalyst or catalyst support towards ORR in an acidic medium.

It is widely recognized that the biggest concern in fuel cells is to improve the performance of the Pt catalysts and one of the main techniques is to have an effective catalyst support. In our research, we report this novel nanostructure, consisting of magnetron sputtered catalyst Pt directly supported on 2D MoS_2_ on individual carbon nanospheres, and its electrochemical performance was investigated under a real single test cell working condition. The test cell is a 5 cm^2^ membrane electrode assembly (MEA) with Pt/MoS_2_/Carbon as the ORR catalyst layer. Due to its excellent water management, it was found to bring out better power output than the Pt sputtered on plain carbon black with reliable electrochemical stability.

## Result and Discussion

### Materials characterization

**Figure 1:**
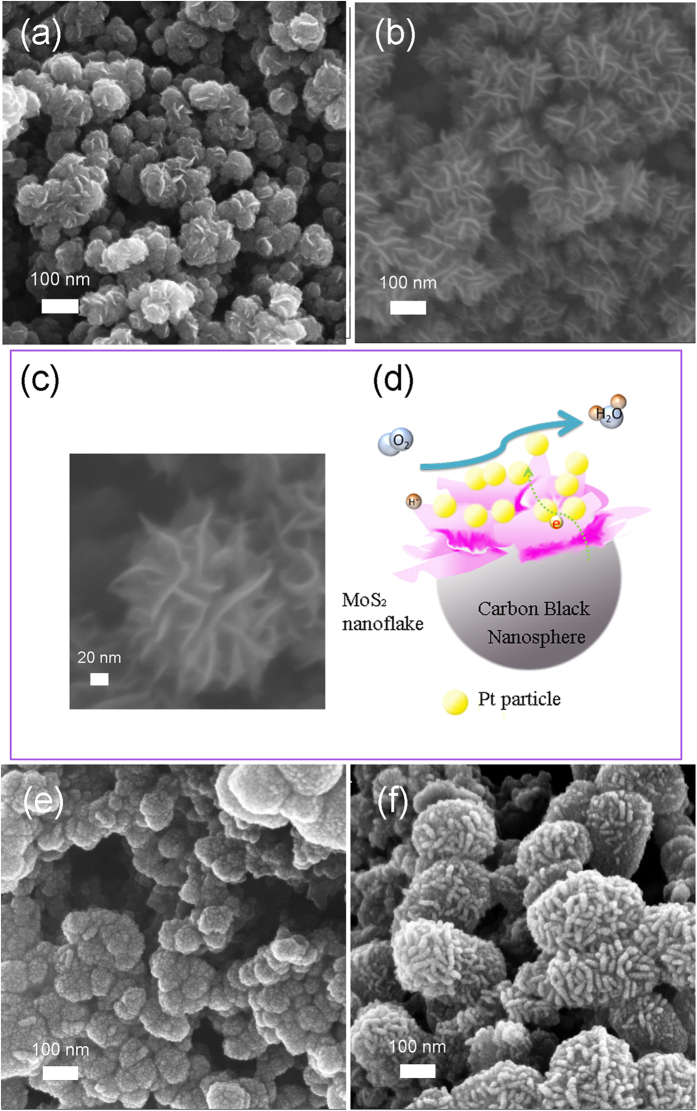
SEM images of 2D MoS_2_ sputtered on carbon black with loading of (**a**) 0.005 mg/cm^2^ and (**b**) 0.01 mg/cm^2^, and the respective samples with Pt deposition of 0.04 mg/cm^2^ in (**e**) and (**f**). There is a small process window in which highly oriented 2D MoS_2_ can be obtained and in Figure 1(**c**), a high resolution image of a single carbon nanosphere with 2D MoS_2_ encapsulating all around the surface. (**d**) Due to the low dimensions, Pt particles line the edges of the 2D MoS_2_ structure giving rise to an increase in effective surface area for electrocatalysis.

Figure 1 shows the SEM images of the after sputtered MoS_2_ with 0.005 and 0.01 mg/cm^2^ MoS_2_ loading, and the respective Pt-MoS_2_ samples with a Pt loading of 0.04 mg/cm^2^. Clearly shown in Fig. 1(a), no feature was observed other than the porous carbon nanosphere (CNS) surface in the sample with 0.005 mg/cm^2^ MoS_2_. However, when the density increased to 0.01 mg/cm^2^ as shown in Fig. 1(b), some crystallites appeared and started to reform into a lamellar “3D” type of nanostructure similar to highly-dense petals perpendicular to the surface; in fact all of the basal planes of the MoS_2_ nanofins are perpendicular to the surface in random orientation. As few MoS_2_ nanofins were observed with 0.005 mg/cm^2^ and instead, it appeared more like in a pre-growth stage; some possibly initial perpendicular layers were observed but not distinct. A high resolution SEM image of a single MoS_2_@CNS is shown in Fig. 1(c) where the distinct individual “wall-like” flakes firmly attached to the sample surface were observed.

**Figure 2:**
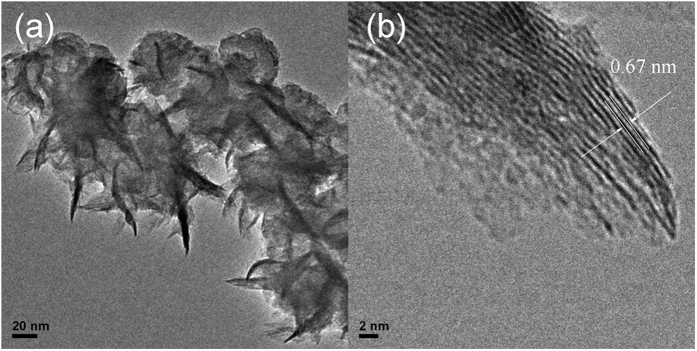
TEM images of (e) 0.01mg/cm^2^ MoS_2_ sputtered on carbon black and (f) HRTEM image on the edge of the 2D MoS_2_, with a clear 0.67 nm crystalline well-aligned structure.

When Pt was sputtered on the 0.005 mg/cm^2^ MoS_2_@CNS, Pt-shells were formed with an average diameter of 10 nm upon CNS, as shown in Fig. 1(e); nevertheless, Pt particles tended to deposit on fringes of the MoS_2_ flakes, resulting possibly in a higher Pt active area that exposed to fuel and electrolyte. As shown in Fig. 1(f), the Pt particles coated the entire surface of the “fins” thus allowing the reaction and Fig. 1(d) an art impression on how it would look like. The TEM images in Fig. 2(a,b) show the CNS and the 2D MoS_2_ layers that grew on the CNS with interlayer distances of 0.67 nm, which is characteristic of MoS_2_.

**Figure 3:**
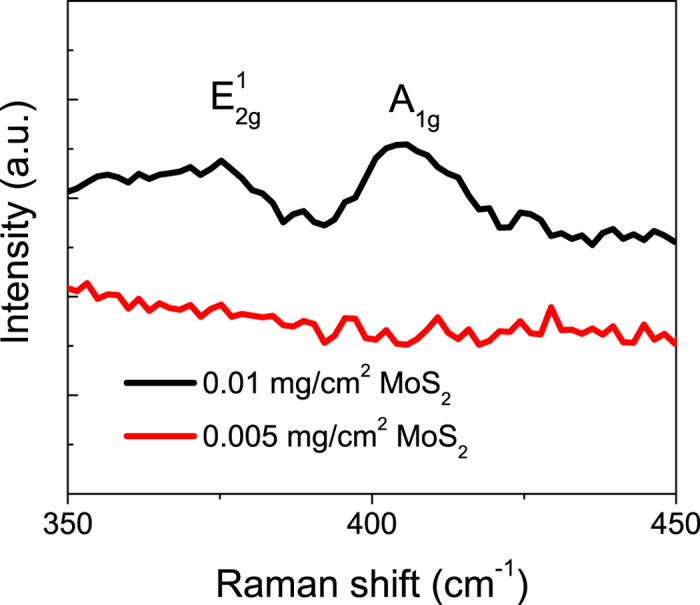
Raman spectra of MoS_2_@CNS confirming the presence of crystalline MoS_2_ on the surface.

**Figure 4:**
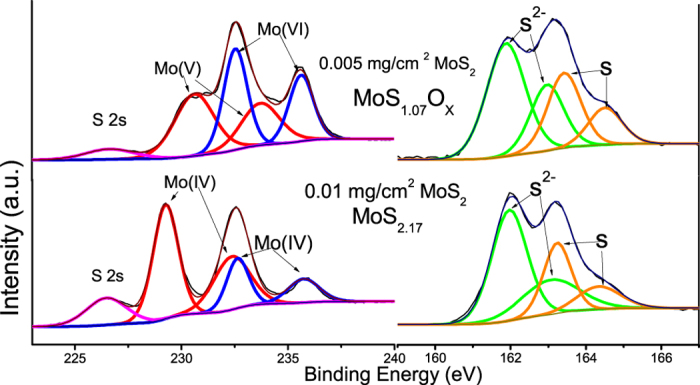
XPS spectra of Mo and S on MoS_2_ with loading of 0.01 mg/cm^2^ and 0.005 mg/cm^2^.

The Raman spectrum of 2D MoS_2_ in Fig. 3 reveals the characteristic peak of MoS_2_ 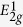 at 375 cm^−1^ and A_1g_ peak at 410 cm^−1^, indicating the existence of the crystalline MoS_2_ structure in 0.01 mg/cm^2^ sample. These results further corroborate the high resolution TEM observation. Moreover, a compositional analysis of the sputtered products was conducted via XPS. As shown in Fig. 4, two doublets of Mo 3d_5/2_ and 3d_3/2_ were observed in XPS spectrum of both concentrations MoS_2_. In the 0.005 mg/cm^2^ sample, the first doublets revealed the core level at 230.6 eV and 233.7 eV with reference to 284.6 eV as the binding energy of C 1s, which shifted towards the lower binding energy of 229.3 eV and 232.4 eV in the 0.01 mg/cm^2^ sample. The binding energy of the former Mo peaks lay between those Mo^4+^ and Mo^5+^ in molybdenum oxide^13^, which implies the existence of a new Mo related phase in the 0.005 mg/cm^2^ sample; on the other hand, the latter doublets reached the energies of 229.3 eV and 232. 4 eV that is consistent with Mo^4+^ in MoS_2_, indicating the occurrence of chemical change in sputtering. The doublets at the higher binding energy corresponded to Mo^6+^ in MoO_3_ as the core level of Mo 3d_5/2_ and 3d_3/2_ centered at 232.5 eV and 235.7 eV^14^,^15^, which predominated in the 0.005 mg/cm^2^ sample but decreased in the relative concentration as the sputtering time increase.

Like the XPS scan of Mo element, all the S spectra exhibited two spin-orbit doublets, i.e. the characteristic peak of the divalent sulfide ion (S^2−^) in MoS_2_ that located at 161.9 eV and 163.1 eV for S 2p_3/2_ and 2p_1/2_, as well as an extra set of S doublets at 163.4 eV and 164.5 eV, suggesting the presence of amorphous Sulfur. Resembling the Mo (VI) ions, the relative intensity of amorphous S peaks experienced noticeable decrease as the sputter duration increase; these phenomena, coupled with the increasing Mo^4+^ and S^2−^ as well as the energy shift of Mo^4+^, suggest the gradual replacement of O atoms by S atoms to form MoS_2_. By the peak deconvolution in XPS spectra, the stoichiometric ratio(S: Mo) was thus determined by the integrated peak area, and the result indicates that the structure of deposited nanofins that prepared by sputtering is close to MoS_2_. It is also noteworthy that the remarkable signal from S^2−^ in MoS_2_ was detected as an indication of Mo-S bonding in the 0.005 mg/cm^2^ sample. However, as the Mo doublets showed binding energies close to its oxide form rather than the sulfurized form, the MoS_2_ produced here is highly defective and Sulphur-deficient, i.e. most of Mo atoms bond to oxygen with minor Mo-S bonding; the conclusion is further corroborated by the stoichiometric ratio (S^2−^: Mo with lower binding energy) calculation, revealing the Mo compound exists in the form of MoS_1.07_O_x_.

To verify the XPS result, a direct TEM observation was performed on the cross-section of the MoS_2_ supported by a Si substrate coupled with EDS spectroscopy (see Supplementary Fig. S1). The MoS_2_ nanofins grew onto the Si wafer with their basal planes perpendicular to the substrate, like the cases that supported by carbon black agglomerates above. The dark layer between the Si and MoS_2_ nanofins was supposed to be the mixture of MoO_3_ and amorphous Sulphur with a thickness of ca.7–8 nm. In addition, the HR-TEM image reveals that all the phases in the base layer were in amorphous form, which agreed well with the absence of respective peaks in Raman spectrum for 0.005 mg/cm^2^ sample. Moreover, the compositional analysis was conducted by the EDS line scan in the parallel direction across base layer and nanofins. The line scan across the nanofins depicts a directly proportional relationship between the Mo and S signals, and the overall S: Mo ratio is 2.07, consistent with the calculated result in XPS section (as illustrated in Supplementary Table S1).

Unlike the nanofins, the relationship between Mo and S is more complicated in the base layer, neither directly nor inversely proportional, thus implying the existence of Mo-S bonding in the molybdenum oxide as well as the relatively independent Sulphur phase.

Combined the observation in XPS, TEM and EDS, it seems that when the sputtering time is short, the originally excited Mo atoms tend to bind with the oxygen species on the carbon surface to form molybdenum oxide while the S atoms prefer to form S-S bonds rather than interact with Mo atoms, thus producing a negligible amount of MoS_2_. As the sputtering proceeds, the arrival of more S atoms on the surface fosters the replacement of O atoms in MoO_2_ to yield MoS_2_. Subsequently, the MoS_2_ nanofins start to grow almost exclusively in its crystalline form.

In terms of the preferential-grown MoS_2_ during sputter deposition, the similar structures, i.e. randomly-oriented MoS_2_ lamellae with voids in between were fabricated by RF sputtering and the growth mechanism has been extensively discussed^16^,^17^,^18^,^19^,^20^,^21^,^22^. First we consider the crystalline structure of MoS_2_: individual MoS_2_ layers are stacked in a staggered arrangement along the c-axis with the weak van der Waals interactions between the sheets of sulfide atoms. Therefore the surface energy of basal planes is much lower than other planes and in general, MoS_2_ preferably grows in 2D basal planes in contact with the surface of the substrate that minimizes the surface energy. The theory agrees well with the beginning growth stage of the sputter MoS_2_ in previous studies^17^,^20^; however, many distinguishable vertical-aligned MoS_2_ nanofins were subsequently observed branching from the basal planes. The researchers proposed that due to the structural anisotropy, the rate of the atomic diffusion along the layers through the van der Waals gap is much faster than the diffusion across the layer gap, thereby making the growth velocity greater in the [uv0] direction than perpendicular to layer. In addition, the outer planes of the layers (Sulphur atoms) are chemically nearly saturated and, therefore, weakly reactive. If lamellae begin to grow perpendicular to the interface, an incident atom will tend to bind itself to one of their reactive edge sites, rather than to the inert surface of the top parallel layer. At edge sites, a mobile adatom can readily diffuse and attach onto a proper site, whereas the basal growth direction requires the sequential addition of an appropriate adatom species, which is a more complicated (and kinetically slower) process. Therefore, the selectively deposition coupled with the preferential diffusion leads to the overwhelming growth of vertical lamellae over the horizontal type, creating voids typical of sputtered thin films. This point of view can explain the phenomenon in our studies, although no basal MoS_2_ planes are detected between the vertical nanofins and the substrate possibly due to the impurity and humidity on surface^17^.

In addition to Mo and S element, the determination of Pt chemical state was carried out by means of spectrum deconvolution using a Gaussian/Lorentzian shape line modified by an asymmetric function. The Pt 4f_7/2_ core level revealed a binding energy of 71.1 eV in all samples with the sputter Pt catalyst, with reference to 284.6 eV as the binding energy of C 1s peak (see Supplementary Fig. S2). This result substantially suggested that the sputter-deposited Pt catalyst is mostly in the pure metallic state.

**Figure 5:**
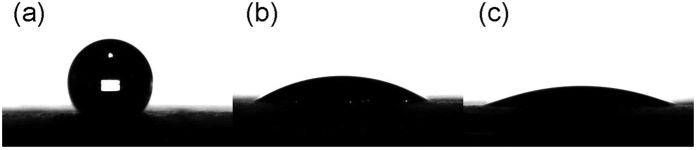
Contact angle test of GDL without coating (**a**) and with (**b**) 0.005 mg/cm^2^ MoS^2^, (**c**) 0.01 mg/cm^2^ MoS_2_.

Apart from the microstructure and composition analysis of the sputtered MoS_2_ above, the surface property of the MoS_2_-coated electrodes was also explored, including the hydrophobic property and porosity. As shown in Fig. 5, the conventional GDL presented an average contact angle of 131.82 due to the hydrophobic binder PTFE that mixed with the carbon black agglomerates. In contrast, the as-deposited MoS_2_ coating altered significantly the hydrophobicity of electrode surface, yielding a reduction in contact angle that ranges from 32.7 to 21.4 as the MoS_2_ loading increases from 0.005 mg/cm^2^ to 0.01 mg/cm^2^. The high wettability of the MoS_2_ modified samples might spring from two aspects, i.e. the nanofins and the base layer. On one hand, the edges of MoS_2_ planes is supposed to be extremely hydrophilic^23^,^24^ due to their high specific free energy (SFE); hereby, the surface texture with vertical oriented (001) planes yields massive exposed edges that in turns increases SFE. On the other hand, the base layer that consist of amorphous MoO_3_ and Sulphur forms a shell on top of the PTFE coated carbon black agglomerates, and the wettability of shell composition counteracts the effect of the hydrophobic binder. Moreover, the effect of MoS_2_ coating on the electrode porosity was evaluated by the BET measurement; the result shows a similar BET surface area of ca. 3.3 m^2^/g between the conventional GDL and the 0.005 mg/cm^2^ MoS_2_ coated electrode, yet the MoS_2_ nanofins enhanced the surface roughness as observed in SEM images, thereby inevitably increasing the BET area, which is 3.5 m^2^/g in the 0.01 mg/cm^2^ MoS_2_ modified electrode.

### Catalytic activity

**Figure 6:**
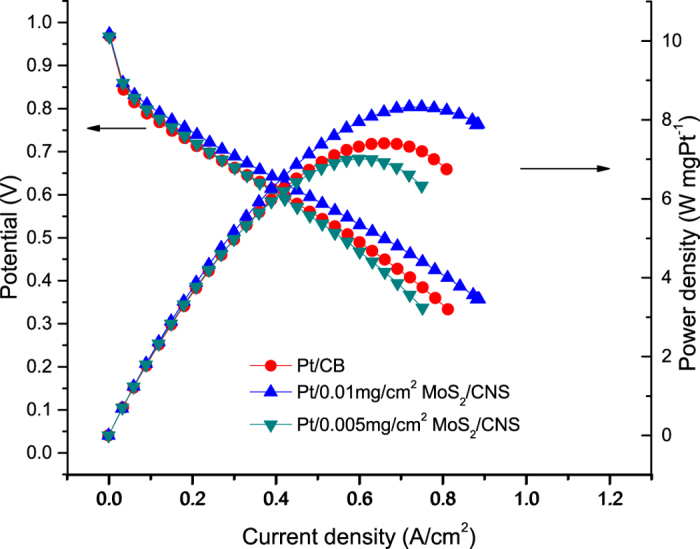
Polarization curves of a variety of 2D Pt/MoS_2_ modified electrodes with the same Pt loading of 0.04 mg/cm^2^ on the cathode.

The effectiveness of this unique microstructure for PEM fuel cell applications was proven by depositing these structures uniformly across a 5 cm^2^ large electrode and integrating it through the fabrication of a membrane electrode assembly and finally, assembling in a PEM fuel cell test unit. Figure 6 shows the polarization curves of the Pt/MoS_2_ modified electrodes with sputtered Pt on pure carbon black (CB) as reference under the same Pt loading of 0.04 mg/cm^2^ on the cathode. As observed, Pt/0.01 mg/cm^2^ MoS_2_ sample realized significantly more prominent Pt utility than the reference Pt/CB sample, yielding an approximate 10% increase in power output. However, in the case when the “fins” of the 2D MoS_2_ were not spread (as in 0.005 mg/cm^2^), the power output was about 5% lower than the reference Pt/CB sample.

**Figure 7:**
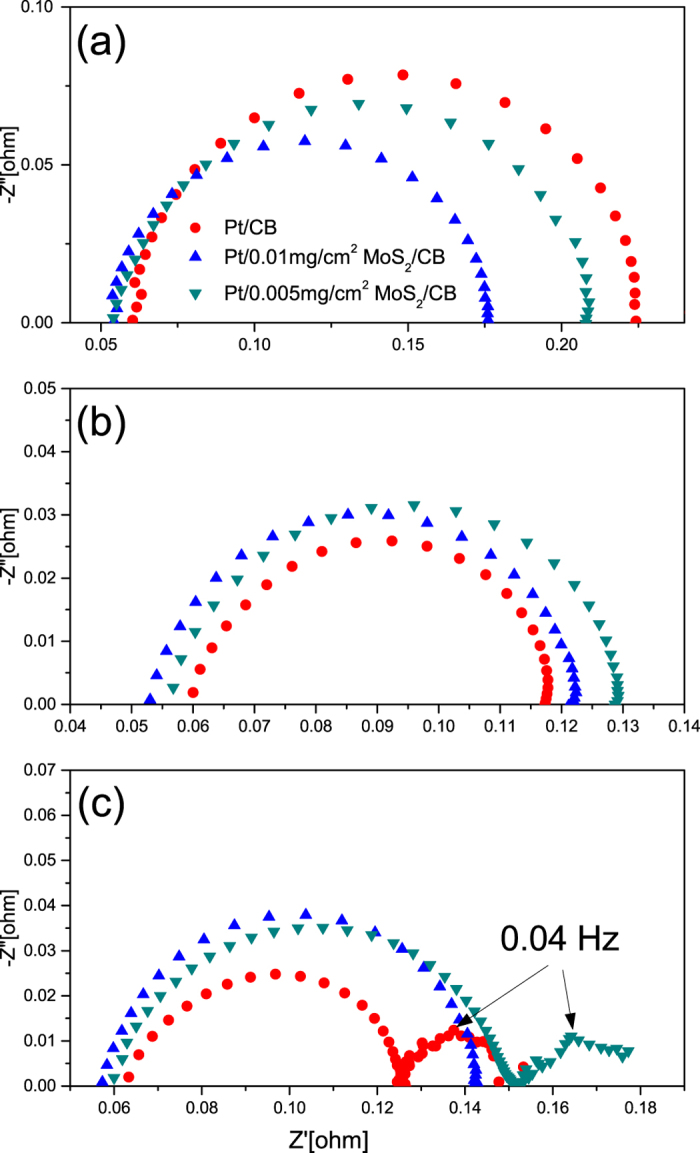
Nyquist spectra of MEAs with Pt/CB and Pt/MoS_2_/CNS based cathodes at (**a**) 0.8 V, (**b**) 0.6 V and (**c**) 0.4 V.

To further understand the cell performance observed in the MoS_2_ modified electrodes, electrochemical impedance spectroscopy (EIS) was employed to evaluate the transport properties of these samples. As shown in Fig. 7(a), the spectra at 0.8 V was appeared to be a semicircle arc that originated from the charge transfer resistance R_ct_ and the double layer capacity C_dl_. All the three electrodes exhibited similar intercept at high frequency that reveals similar electrolyte resistance R_el_, which permits the easy comparison of charge transfer resistance based on the intercept at low frequency terminate.

Interestingly, the Pt/0.01 mg/cm^2^ MoS_2_ electrode showed the smallest R_ct_, which might stem from the self humidifying process in the highly hydrophilic surface at low current densities, favoring the ORR kinetic at the electrode-membrane interface as reported in literature^25^. The advantage of humid environment for the ORR reaction could also explain the lower R_ct_ of Pt/0.005 mg/cm^2^ MoS_2_ electrode compared to the hydrophobic reference electrode at the stage where the ORR kinetic controls.

When the cell potential dropped to 0.6 V, the R_ct_ loop shrunk for all three electrodes as shown in Fig. 7(b). It can be attributed to the increasing driving force for ORR as the cathode overpotential increases. Moreover, the intercepts at high frequency terminate of the reference electrodes as well as the 0.005 mg/cm^2^ sample shifted positively, which indicates the increase in electrolyte resistance; given the higher current density, the R_el_ variation might be caused by the dehydration of Nafion membrane and/or electrolyte. As the enlarged current densities means the faster chemical reaction and osmotic drag, i.e. the faster product of water at cathode side, the drying-out of membrane is thus expected possibly to occur at the anode side which will be discussed further in the next section. In addition, the R_ct_ of the Pt-MoS_2_ catalysts exceeded that of the Pt/CB catalyst in this current region, reflecting that the MoS_2_ modified catalysts lost their advantage over the reference electrode in terms of Pt catalyst utility; this phenomenon might be explained by the low electrical conductivity of MoS_2_ nanofins and the base MoO_3_ layer that retard the decrease of charge transfer resistance when the overpotential augments.

As the cell potential decreases to 0.4 V, i.e. the mass transport overpotential domain, the radius of the high frequency loop increased among all the MEAs, which indicates the emergence of another relaxation process with similar time constant to the charge transfer process. One possible explanation might be the oxygen diffusion resistance R_od_ inside the sputtered catalyst layer. As the sputter Pt particles are located mainly at the electrode-membrane interface, the dense accumulation of produced water in the narrow area might occur and thus obstruct the passage of Oxygen gas to reaction sites, which would further aggravated by the hydrophilic MoS_2_ layers. However, due to the similar time constants for the two processes, the contribution of R_ct_ and R_od_ to the enlarged high frequency loop is hard to be explicitly resolved; therefore the impact of MoS_2_ coating is still not quantitatively determined on the individual R_od_ at the mass-transport control region.

Another change of the Nyquist plots at 0.4 V is the new low frequency loop that appeared in all the impedance plots except of the Pt catalysts that supported on MoS_2_ nanofins; this semicircular arc could be attributed to the water transport resistance R_wt,_ which seems to develop into a major limiting factor that cause severe potential drop. In contrast to the R_ct_ and R_od_, the water transport limitation has been less extensively studied; moreover, the origin of water transport resistance is more controversial^26^,^27^,^28^. Herein, the additional loops at low frequency revealed the identical characteristic frequency of 0.04 Hz in the different electrodes, which corresponds the Paganin’s conclusion^26^ that the process is attributed to the water transportation in the membrane and the respective time constant is merely membrane-related and independent of the cathode microstructure or composition. In addition, a more quantitative analysis on the mass transport properties of the MEAs with N117 membrane could be conducted based on the assumption that water diffusion across membrane and electrolyte is the limiting step. It is known that the characteristic frequency for the finite diffusion impedance can be determined by Equation (1)^29^,


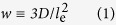


where *D* is the diffusion coefficient and *l*_*e*_ is the effective diffusion length. Taking 7 × 10^−6^ cm^2^ s^−1^ as the diffusion coefficient of water in fully-hydrated Nafion^30^ and 0.04 Hz as the characteristic frequency for diffusion process, the length for water backtransport was thus estimated to be 91.4 μm. The results are in excellent agreement with the membrane thickness of ca.90–100 um that observed in SEM image of MEA cross-section (see Supplementary Fig. S3); it is confirmed that the mass transport is mainly controlled by the water diffusion across membrane. Nevertheless, the difference in magnitude of R_wt_ is supposed to spring from the different cathode electrodes and their current density at the certain potential; based on our assumption that R_wt_ depends on membrane dehydration, it’s reasonable to expect a larger value of R_wt_ when the current density increases and thus more proton-hydrating water transport from the anode side. However, given the similar current densities@0.4 V for three MEAs, their cathode property determines the different R_wt_ value. Particularly, no second arc appeared in Pt supported on nanofins catalysts, which possibly indicates that the existence of MoS_2_ nanofins seems to retard or extinguish the water diffusion resistance. A possible explanation for the absence of diffusion loop in the Pt-MoS_2_ catalysts is the porous MoS_2_@CNS structure that differ it primarily from the hydrophilic Pt-MoO_3_ (0.005 mg/cm^2^), which might yield better contact between catalyst and electrolyte to facilities the diffusion process of the built-in water at the membrane-electrode interface.

### Electrochemical stability

**Figure 8:**
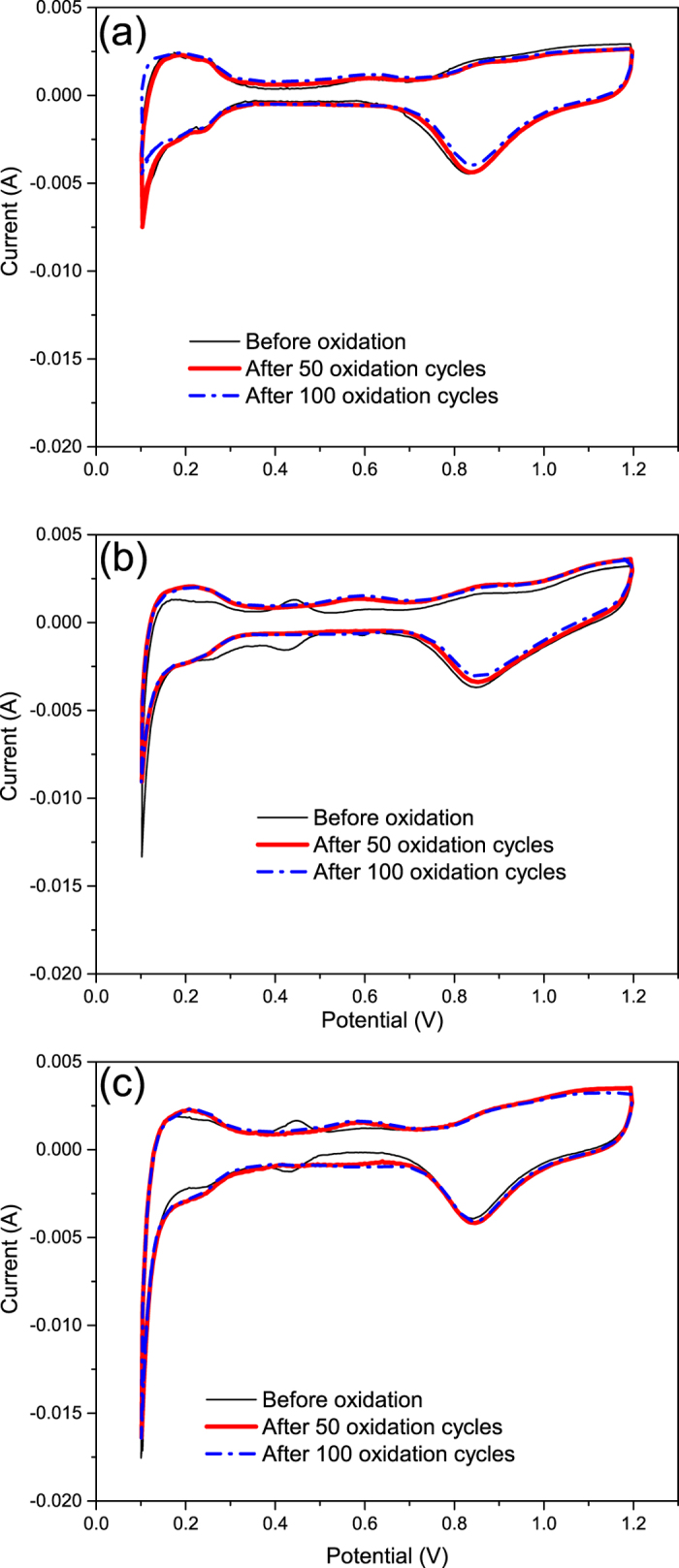
Cyclic voltammograms of (**a**) Pt/CB, (**b**) Pt/0.01 mg/cm^2^ MoS_2_/CNS, (**c**) Pt/0.005 mg/cm^2^ MoS_2_/CNS samples before and after 50 and 100 oxidation cycles.

Accelerated degradation test (ADT) utilizing oxidation cycling was performed on all 3 samples to study their robustness in the PEM fuel cell test cells, simulating actual working environment. Figure 8 shows the *in situ* cyclic voltammograms of all the MEAs under a scan rate at 50 mV/s before and after 50 and 100 cycles of oxidation. Before oxidation, two pairs of typical characteristic peak for Pt metal were observed in each voltammogram, including the hydrogen adsorption/desorption peaks that centered at 0.2 V as well as the Pt oxidation/reduction peak located around 0.8 V. The MoS_2_-modified electrodes yielded an extra pair of peak at around 0.43 V that indicated the transformations of Mo species, which has been reported in previous studies on Mo-related catalyst in terms of ORR at acidic medium, e.g. Pt-Mo alloy^31^,^32^, Pt-MoC_2_^33^,^34^ and Pt-MoO_3_^35^. As a result, the current below 0.4 V originated from the overlap of the electrochemical activity of Pt with that of Mo, and the respective ECSA calculation was not accurately determined with the inference of Mo on the hydrogen adsorption/desorption peak area. After 10 potential cycles, the active area of Pt catalyst readily recovered from Mo masking effect associated with the disappearance of the additional pair of Mo-related peaks, implying the adsorption of Mo oxygenated group on Pt surface in the presence of Mo redox reaction as well as the subsequent Mo dissolution. During the oxidation, the sputter Pt catalyst showed visibly high resistance to corrosion, as only a slight change was observed in cyclic voltammograms tested before and after 50 and 100 oxidation cycles. Furthermore, the prominent stability of sputter Pt was not undermined by the additional MoS_2_ support given their similar aging performance in comparison to the Pt/CB electrode. One of the reasons might be the larger Pt particle size in the sputter coated samples that is less sensitive to oxidation with low active area and mobility during the supporting carbon corrosion. More evidence of stability for the Pt/MoS_2_ catalyst could be provided by the current response to the high potential during the ADT. Supplementary Fig. S4 illustrates the first 10 cycles of oxidation for the Pt/MoS_2_ catalysts as well as the reference electrode. It is noteworthy that the Pt/MoS_2_ catalyst yielded the oxidation current with similar or less amplitude and change in comparison with the Pt/CB catalyst under the high potential of 1.4 V, which suggests the robustness of CNS that encapsulated by MoS_2_ nanofins.

To elucidate the Mo activity under the oxidation test, a direct and clear visualization was realized by the CVs of the Pt/0.01 mg/cm^2^ MoS_2_ based electrode under the potential cycling between 0.2 V and 1.2 V with the scan rate of 50 mV/s (as shown in Supplementary Fig. S5). The cathodic and anodic current at 0.43 V experienced a gradual decrease over the enduring oxidation-reduction cycles. By contrary, the hydrogen adsorption/desorption behavior on the Pt catalyst surface became more active as their corresponding current exhibited a remarkable increase as the CVs swept, indicating the recovery of catalytic ECSA from the Mo interference. The change in Pt and Mo-related current simultaneously reflect the Mo dissolution into electrolyte from Pt/MoS_2_ catalyst. In an attempt to quantify the stability of the sputtered MoS_2_, the peak currents at 0.43 V were normalized by the current maximum at the initial cycle, and then plotted out *vs.* the cycle numbers (as shown in Supplementary Fig. S6). Under PEMFC working conditions, the peak current plunged sharply at the beginning 100 CV cycles that followed by a milder decease in the sequential 200 rounds. More importantly, the post-CV SEM observation of Pt/MoS_2_ catalyst (see in Supplementary Fig. S7) confirms the corrosion resistance of MoS_2_@CNS; the Pt 3D network still attached tightly to the surface of CNS. Although the status of MoS_2_ was hardly detected due to the coverage of Pt nanoparticles, it is reasonable to speculate that the lamellar structure of MoS_2_ nanofins partly survived under the corrosive potential cycling, given the intimate interaction between Pt nanoparticles and carbon black support.

## Conclusion

The novel MoS_2_@CNS structure was fabricated by a combined method of magnetron sputtering and ink spray, which served as the catalyst support for PEM fuel cell cathode. The microstructure and surface property was investigated as well as their electrochemical activity and stability. The Pt supported on 0.01 mg/cm^2^ MoS_2_ demonstrated an enhanced electrochemical activity in a 5 cm^2^ PEM fuel cell single cell test in comparison with the sputter Pt that deposited directly on CNS. The improved catalytic performance might be correlated with the eminent water management out of the MoS_2_ nanofilm texture as humid retention at low current region and flood removal at high current region.

## Method

### Preparation of MEAs

In this study, the fuel cell tests were conducted on a 5 cm^2^ membrane electrode assembly that prepared by thin-film method^36^. The carbon paper TGPH090 (Toray Corp.) was coated with a porous gas diffusion layer (GDL) that was composed with a mixture of carbon black (VXC-72R) and Polytetrafluoroethylene (PTFE, Sigma Aldrich) (weight ratio of C to PTFE was 70:30; carbon loading was 1.4 mg cm^−2^). Then an ultrathin MoS_2_ layer was coated upon GDL by a radio frequency magnetron sputtering system (Denton Discovery-18), which was followed by Pt nanoparticles deposition as catalyst with a loading of 0.04 mg/cm^2^. Meanwhile, the Pt catalyst layer was deposited on the carbon black GDL to fabricate a reference electrode. Besides, the standard electrode was prepared by applying the suspension of 20% Pt/VXC-72R (ETEK) and 5% Nafion^@^ perfluorinated resin solution (Sigma Aldrich) on the gas diffusion layer, which serves as anode in all the MEAs with the Pt loading of 0.2 mg/cm^2^ and Nafion loading of 0.1 mg/cm^2^. By the hot press, the as-prepared electrodes and Nafion 117 membrane as electrolyte completed the MEA fabrication. Prior to the assembly process, the sputter Pt-based electrodes, i.e. Pt/CB and Pt/MoS_2_ series, were spread with 6 uL of 5% Nafion perfluorinated resin solution via the air brush, in order to improve the ionic conductivity between the Nafion membrane and the electrodes.

### Characterization of Pt/MoS_2_ electrodes

The MEAs performance was evaluated by a single cell test station 890B (Electronchem Corp.) when the experiment details can been seen elsewhere^37^. *In situ* electrochemical impedance spectra (EIS) (Autolab 302N) was employed at 0.8, 0.6 and 0.4 V vs. DHE from 0.01 Hz to 10000 Hz. Cyclic Voltammogram were recorded from 0.2 V to 1.2 V vs. DHE at a rate of 50 mV/s before and after every 10 oxidation cycles of an accelerated degradation test (ADT), which was performed based on the oxidation potential cycling between 0.6 and 1.4 V vs. DHE.

In addition, the microstructure of MoS_2_ deposited electrodes was characterized by scanning electron microscopy (SEM, JEOL JSM-6700F) and transmission electron microscopy (TEM, JEOL JEM-2010). The composition of MoS_2_ nanofilm was further clarified by a Raman spectroscope (514.5 nm green laser, Renishaw 2000) and high resolution x-ray photoelectron spectroscope (XPS) (Axis Ultra DLD) using a 400 W monochromated AlKα source with a base pressure of 1 × 10^−9^ torr. The pass energy of 20 eV was used for narrow scan. An ASAP-2000 BET characterization system (Micromeritics Instrument Corp.) was used to measure the surface area of MoS_2_ coated electrodes. The wettability of MoS_2_ coated electrodes was evaluated by a contact angle analyzer (VCA optima, AST Product Inc.).

## Additional Information

**How to cite this article**: Hu, Y. and Chua, D.H.C. Synthesizing 2D MoS_2_ Nanofins on carbon nanospheres as catalyst support for Proton Exchange Membrane Fuel Cells. *Sci. Rep.* **6**, 28088; doi: 10.1038/srep28088 (2016).

## Supplementary Material

Supplementary Information
