# Supplementary material for: Synthesizing 2D MoS2 Nanofins on carbon nanospheres as catalyst support for Proton Exchange Membrane Fuel Cells
Source: Sci Rep. 2016 Jun 15;6:28088. doi: 10.1038/srep28088 (PMC4908422; doi:10.1038/srep28088)
Supplement: Supplementary Information [file srep28088-s1.pdf]

Synthesizing 2D MoS_2 Nanofins on carbon nanospheres as catalyst support for Proton Exchange Membrane Fuel Cells

Yan Hu and Daniel H.C. Chua *

Table S1 Mo and S atomic ratios in EDS line scan.

	Mo Atomic%	S Atomic%	S:Mo ratio
Base layer	3.69	11.33	3.06
Nanofins	6.08	12.57	2.07

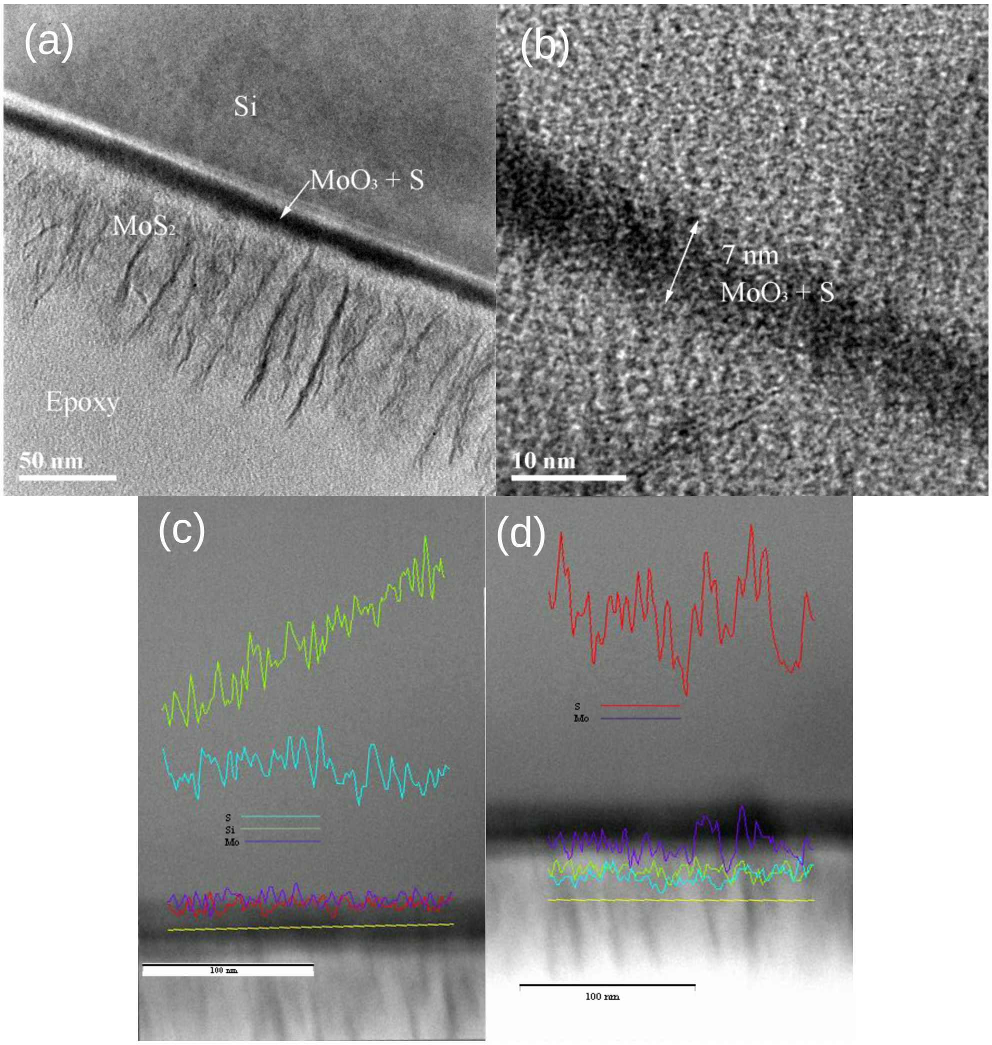

Fig. S1 (a) Cross-section TEM of 0.01 mg/cm^2 MoS_2 deposited on Si substrate and (b) HRTEM image of base layer; EDS line scan of (c) base layer (Cyan line: S, Green line: Si, Violet line: Mo) and (d) nanofins (Red line: S, Violet line: Mo).

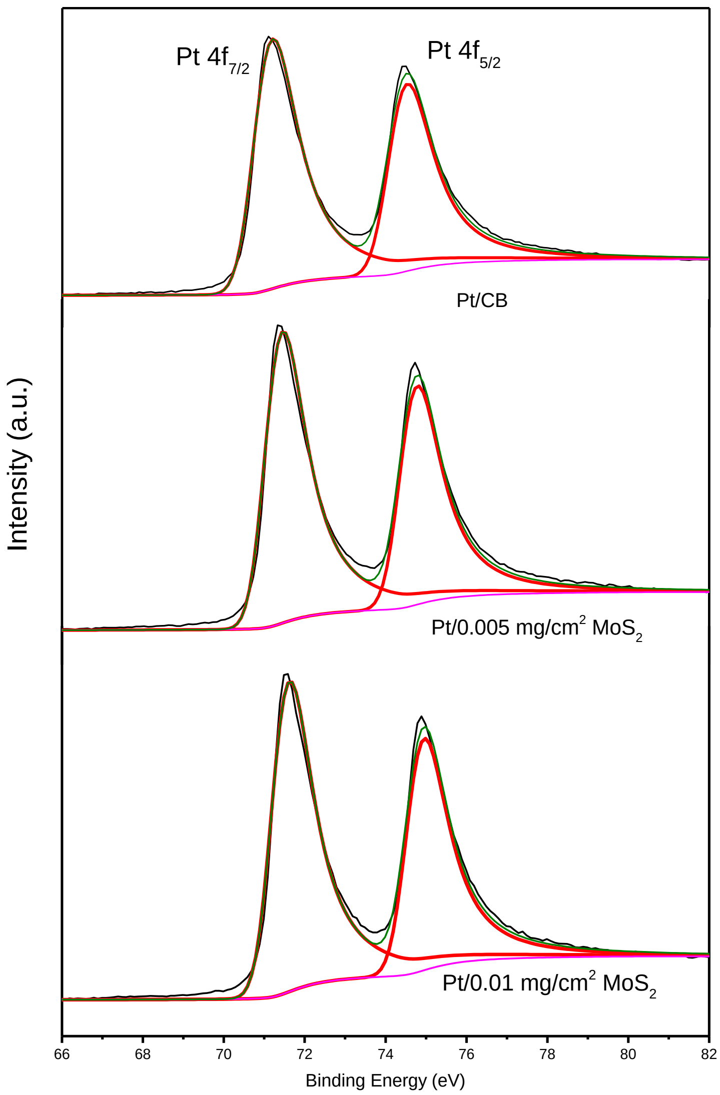

Fig. S2. XPS spectra of Pt 4f core level region of Pt supported on carbon black and MoS_2 with loading of 0.005 mg/cm^2, 0.01 mg/cm^2.

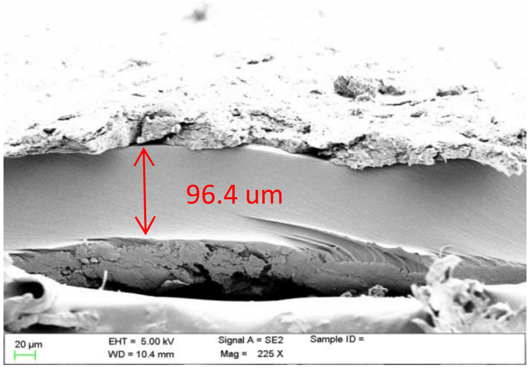

Fig S3 SEM image of cross-section for the MEA with N117 membrane

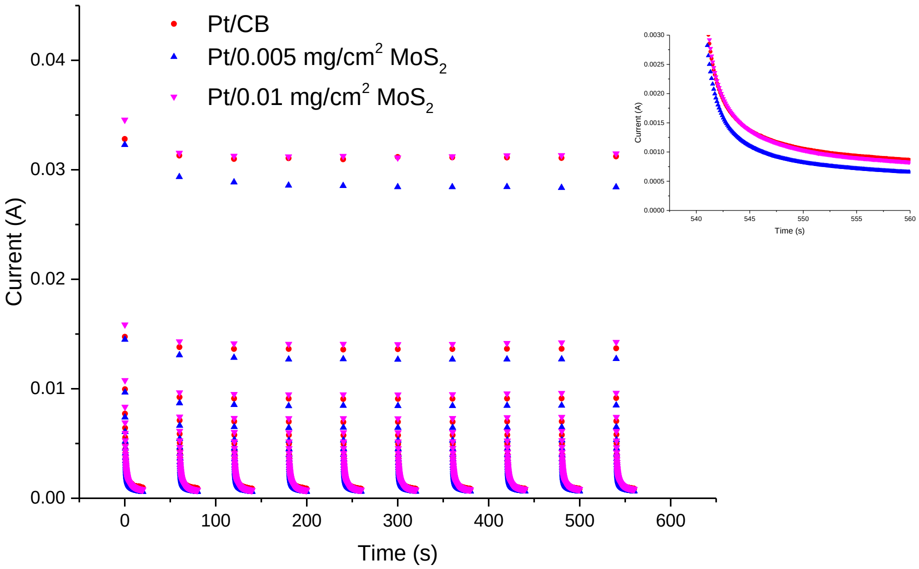

Fig. S4 The first 10 oxidation cycles for Pt/MoS_2 catalysts and reference (inset: current at 1.4 V for 10th oxidation cycle).

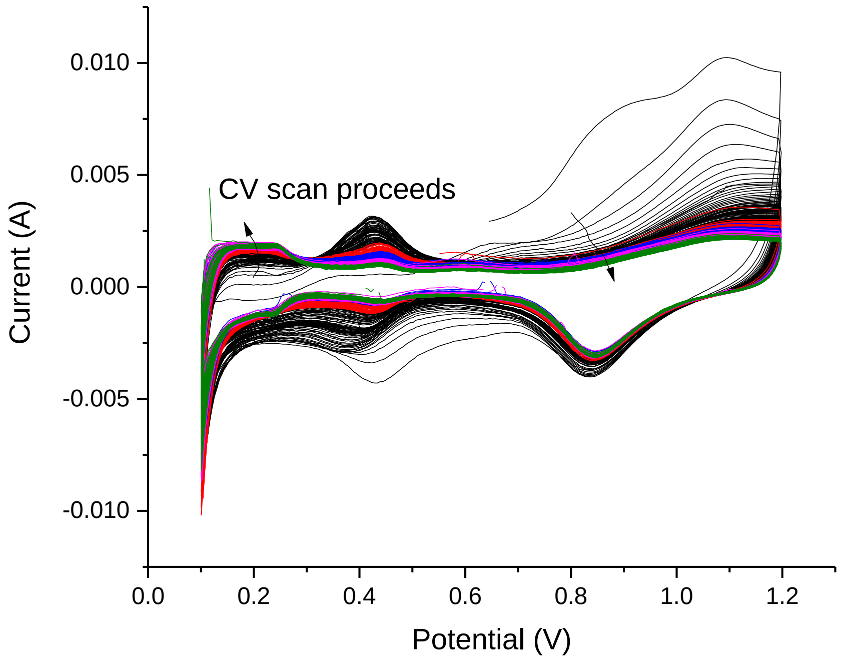

Fig. S5 Cyclic voltammograms of Pt/0.01 mg/cm^2 MoS_2@CNS from 1st to 320th round scans.

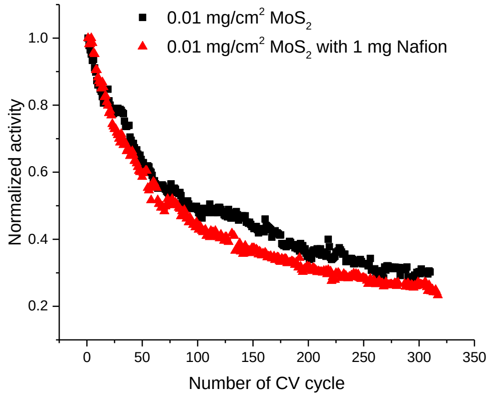

Fig. S6 Normalized activity of Mo peak current@ 0.43 V in the respective CV for Pt/MoS_2 catalyst.

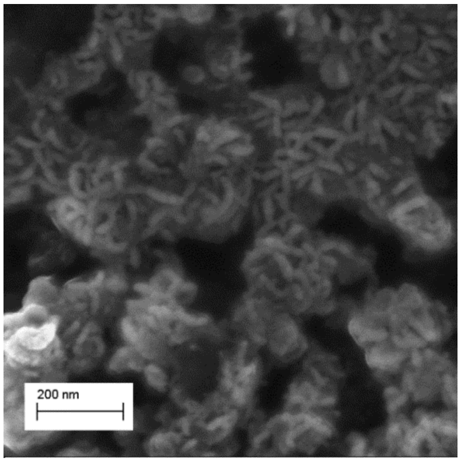

Fig. S7 SEM images of post-ADT $Pt/MoS_2/CNS$ catalyst.
